# S09-2: Physical activity in the Irish vision impaired and blind population

**DOI:** 10.1093/eurpub/ckae114.237

**Published:** 2024-09-26

**Authors:** Lisa Flynn

**Affiliations:** Sport Ireland, Dublin, Ireland; Vision Sport Ireland, Ireland; Institute of Education; Dublin city University, Dublin, Ireland

## Abstract

**Purpose:**

Physical activity (PA) is known to improve physical and mental health. Research suggests that adults and children who are vision impaired (VI) have reduced levels of PA compared to their sighted peers. In the Irish context, census data suggests that up to 40% of people with VI struggle to participate in leisure and social activities. This project aims to understand PA levels, and the barriers/motivators to participation, in the Irish VI population.

**Methods:**

Questionnaires were used to assess self-reported PA, sports participation, perceived barriers to participation and mental health/well-being levels using validated and reliable tools (CSPPA, Kidscreen-10, HSBC survey) ​(Booth et al., 2001; Woods CB et al., 2018)​. Fundamental movement skills (FMS) and VO2 max were assessed in VI children using the TGMD-3 and the Queen’s College Step ​(Maciej Serda et al., 2018)​.

**Results:**

The children’s results (n = 61, mean 12.2 years (range 6-16 years)) showed just 12.7% were active more than 30 mins daily each week. Barriers in this cohort included fear of injury (82%) and a lack of confidence (63%). Further analysis (n = 59, age 12.34 ± 2.40) indicate that just 52.2% of females and 68.6% of males were in the healthy fitness zone according to their VO2 max. Worst performed FMS included two-hand strike, kick and throw with 30.5%, 22% and 30.5% achieving mastery/near-mastery respectively.

The adult population (n = 258, f = 138, m = 173, non-binary = 16, mean = 25 years (range 17-78)) showed the most popular forms of PA included gym classes (18.49%), walking (15.96%) and running (9.80%). Lack of transport (25.8%) and lack of accessible facilities (15.2%) were the most commonly cited barriers to PA.

**Conclusion:**

PA levels were low in both the youth and adult VI populations when compared to the general population. Future research should focus on further exploring the barriers to participation.

**Support/Funding Source:**

Vision Sport Ireland, Institute of Education, Dublin City University, Sport Ireland.

